# Supplementary material for: Application of quantile regression to examine changes in the distribution of Height for Age (HAZ) of Indian children aged 0–36 months using four rounds of NFHS data
Source: PLoS One. 2022 May 27;17(5):e0265877. doi: 10.1371/journal.pone.0265877 (PMC9140296; doi:10.1371/journal.pone.0265877)
Supplement: Attachment — Submitted filename: PONE-D-21-11573_reviewer_JAC.docx [file pone.0265877.s001.docx]

PLOS ONE

Application of Quantile Regression to examine changes in the distribution of Height for
Age (HAZ) of Indian children aged 0-36 months using four rounds of NFHS data.
--Manuscript Draft--

| Manuscript Number: | PONE-D-21-11573 |
| --- | --- |
| Article Type: | Research Article |
| Full Title: | Application of Quantile Regression to examine changes in the distribution of Height for  Age (HAZ) of Indian children aged 0-36 months using four rounds of NFHS data. |
| Short Title: | Quantile Regression to see change in Ht for Age (HAZ) of 0-36 M Indian children :four  rounds of NFHS data |
| Corresponding Author: | Mendu Vishnu Vardhana Rao, PhD  IRMS: National Institute of Medical Statistics  New Delhi, Delhi INDIA |
| Keywords: |  |
| Abstract: | Background In India, it has been observed that the prevalence of stunting among  under-five children decreased, but the prevalence is still alarmingly high. In previous  studies, traditional (linear and logistic) regression analyses were used, and these  analyses were limited to encapsulated cross-distribution variations. The objective of  the study was to examine how the different determinants are heterogeneous in various  percentiles of height for age (HAZ) distribution. |
| Order of Authors: | Mendu Vishnu Vardhana Rao, PhD |
| Additional Information: |  |
| Question | Response |
| Financial Disclosure  Enter a financial disclosure statement that describes the sources of funding for the  work included in this submission. Review the [submission guidelines](http://journals.plos.org/plosone/s/submission-guidelines#loc-financial-disclosure-statement) for detailed  requirements. View published research  articles from [PLOS ONE](http://journals.plos.org/plosone/) for specific  examples.  This statement is required for submission and will appear in the published article if the submission is accepted. Please make sure it is accurate. | This work is a PhD work of a my student and no specific budget is available for publication charges |

*Powered by Editorial Manager® and ProduXion Manager® from Aries Systems Corporation*

| Unfunded studies  Enter: The author(s) received no specific funding for this work.  Funded studies  Enter a statement with the following details:  Initials of the authors who received each  award  •Grant numbers awarded to each author  •The full name of each funder  •URL of each funder website  Did the sponsors or funders play any role in the study design, data collection and  analysis, decision to publish, or preparation of the manuscript? | |  |
| --- | --- | --- |
| • | NO - Include this sentence at the end of  your statement: The funders had no role in study design, data collection and analysis, decision to publish, or preparation of the  manuscript. |  |
| •YES - Specify the role(s) played.  * typeset | |  |
| Competing Interests  Use the instructions below to enter a  competing interest statement for this  submission. On behalf of all authors,  disclose any [competing interests](http://journals.plos.org/plosone/s/competing-interests) that  could be perceived to bias this  work—acknowledging all financial support and any other relevant financial or non-  financial competing interests.  This statement is required for submission and will appear in the published article if the submission is accepted. Please make sure it is accurate and that any funding  sources listed in your Funding Information later in the submission form are also  declared in your Financial Disclosure  statement.  View published research articles from [PLOS ONE](http://journals.plos.org/plosone/) for specific examples. | | This is a PhD thesis work of a student. There is no budged ear marked for publication charges |

•

•

*Powered by Editorial Manager® and ProduXion Manager® from Aries Systems Corporation*

| NO authors have competing interests  Enter: The authors have declared that no competing interests exist.  Authors with competing interests  Enter competing interest details beginning with this statement:  I have read the journal's policy and the  authors of this manuscript have the following competing interests: [insert competing  interests here]  * typeset |  |
| --- | --- |
| Ethics Statement  Enter an ethics statement for this  submission. This statement is required if the study involved:  •Human participants  •Human specimens or tissue  •Vertebrate animals or cephalopods •Vertebrate embryos or tissues  •Field research  Write "N/A" if the submission does not require an ethics statement.  General guidance is provided below.  Consult the [submission guidelines](http://journals.plos.org/plosone/s/submission-guidelines#loc-guidelines-for-specific-study-types) for  detailed instructions. Make sure that all  information entered here is included in the Methods section of the manuscript. | National Institute of Nutrition  National Institute of Medical statistics |

*Powered by Editorial Manager® and ProduXion Manager® from Aries Systems Corporation*

| Format for specific study types  Human Subject Research (involving human  participants and/or tissue)  Give the name of the institutional review  board or ethics committee that approved the study | |  |
| --- | --- | --- |
| •  • | Include the approval number and/or a statement indicating approval of this research  Indicate the form of consent obtained  (written/oral) or the reason that consent was not obtained (e.g. the data were analyzed  anonymously) |  |
| Animal Research (involving vertebrate  animals, embryos or tissues)  Provide the name of the Institutional Animal  Care and Use Committee (IACUC) or other  relevant ethics board that reviewed the  study protocol, and indicate whether they  approved this research or granted a formal  waiver of ethical approval | |  |
| •  •  • | Include an approval number if one was obtained  If the study involved non-human primates, add additional details about animal welfare and steps taken to ameliorate suffering  If anesthesia, euthanasia, or any kind of  animal sacrifice is part of the study, include briefly which substances and/or methods were applied |  |
| Field Research  Include the following details if this study  involves the collection of plant, animal, or  other materials from a natural setting:  •Field permit number  Name of the institution or relevant body that granted permission | |  |
| Data Availability  Authors are required to make all data  underlying the findings described fully  available, without restriction, and from the time of publication. PLOS allows rare  exceptions to address legal and ethical  concerns. See the [PLOS Data Policy](http://journals.plos.org/plosone/s/data-availability) and [FAQ](http://journals.plos.org/plosone/s/data-availability#loc-faqs-for-data-policy) for detailed information. | | Yes - all data are fully available without restriction |

•

•

•

*Powered by Editorial Manager® and ProduXion Manager® from Aries Systems Corporation*

| A Data Availability Statement describing  where the data can be found is required at submission. Your answers to this question constitute the Data Availability Statement and will be published in the article, if  accepted.  Important: Stating ‘data available on request from the author’ is not sufficient. If your data  are only available upon request, select ‘No’ for  the first question and explain your exceptional situation in the text box.  Do the authors confirm that all data  underlying the findings described in their manuscript are fully available without  restriction? | |  |
| --- | --- | --- |
| Describe where the data may be found in full sentences. If you are copying our  sample text, replace any instances of XXX with the appropriate details. | | open source data of govt of India and is available to research scholars |
| •  •  • | If the data are held or will be held in a public repository, include URLs,  accession numbers or DOIs. If this  information will only be available after acceptance, indicate this by ticking the box below. For example: All XXX files are available from the XXX database (accession number(s) XXX, XXX.).  If the data are all contained within the manuscript and/or Supporting  Information files, enter the following: All relevant data are within the  manuscript and its Supporting  Information files.  If neither of these applies but you are able to provide details of access  elsewhere, with or without limitations, please do so. For example:  Data cannot be shared publicly because of [XXX]. Data are available from the  XXX Institutional Data Access / Ethics  Committee (contact via XXX) for  researchers who meet the criteria for  access to confidential data.  The data underlying the results  presented in the study are available  from (include the name of the third party |  |

*Powered by Editorial Manager® and ProduXion Manager® from Aries Systems Corporation*

| • | and contact information or URL).  This text is appropriate if the data are owned by a third party and authors do not have permission to share the data. |  |
| --- | --- | --- |
| * typeset | |  |
| Additional data availability information: | | Tick here if the URLs/accession numbers/DOIs will be available only after acceptance  of the manuscript for publication so that we can ensure their inclusion before  publication. |

*Powered by Editorial Manager® and ProduXion Manager® from Aries Systems Corporation*

Click here to access/download;Manuscript;Quantile Regression Manuscript.doc

**Application of Quantile Regression to examine changes in the distribution of Height for Age (HAZ) of Indian children aged 0-36 months using four rounds of NFHS data.**

Thirupathi Reddy Mokalla^1^, Vishnu Vardhana Rao Mendu^2*^

1Senior Research Fellow, Biostatistics, Public Health Nutrition Division, ICMR-National Institute of Nutrition, Jamai-Osmania, Hyderabad – 500007, Telangana, India Email id:

[thirupathireddy2423@gmail.com](mailto:thirupathireddy2423@gmail.com)

2Scientist G & Director, ICMR-National Institute of Medical Statistics, Department of Health Research, MOHFW, New Delhi-110029, India Email id: [Dr_vishnurao@yahoo.com](mailto:Dr_vishnurao@yahoo.com)

*Corresponding author Vishnu Vardhana Rao Mendu, Email id: [Dr_vishnurao@yahoo.com](mailto:Dr_vishnurao@yahoo.com)

**Authors' contributions**

Thirupathi Reddy M contributed to the data collection, analysis, and manuscript preparation. Vishnu Vardhana Rao M developed the study protocol, secured funds, supervised the study, and guided in manuscript preparation.

**Application of Quantile Regression to examine changes in the distribution of Height for Age (HAZ) of Indian children aged 0-36 months using four rounds of NFHS data.**

**Abstract**

**Background:** In India, it has been observed that the prevalence of stunting among under- five children decreased, but the prevalence is still alarmingly high. In previous studies, traditional (linear and logistic) regression analyses were used, and these analyses were limited to encapsulated cross-distribution variations. The objective of the study was to examine how the different determinants were heterogeneous in various percentiles of height for age (HAZ) distribution.

**Methods**:

This article examined the change in the HAZ distribution of children and examined the relationships between the key covariate's trends and patterns in HAZ among children aged <3 years over a period of 24 years. Four successive rounds of the National Family Health Survey data 1992-93, 1998-99, 2005-06, and 2015-16 were used for analysis. The final study included 206579 children aged <3 years (N= 106136 male, 100443 female). To explain and analyze differences in the HAZ distribution, the lambda-mu-sigma (LMS) method was used [need reference]. Trends in height for age (HAZ) distribution over time were analysed using separate sex- stratified quantile regression (QR). The selected socio-economic, demographic and other predictors were considered for this analysis.

**Results:** The quantile regressions have indicated that mothers who have higher than primary level education were more proactive in mitigating malnutrition among children at the lower end of the distribution. The age, birth order, mother's body-mass-index (BMI) and economic status, among others, were some more determining factors for HAZ. Results of selected quantile regression were estimated at the 5^th^, 10^th^, 25^th^, 50^th^, 75^th^, 90^th^, and 95^th^ quantiles.
**Conclusions:** The outcome of various covariates working differently across the HAZ distribution was suggested by quantile regression. The major discrepancies in different aspects were underlined by socioeconomic and demographic aspects among the Indian population. The heterogeneity of this effect was shown using quantile regression.

**Keywords:** Body Mass Index (BMI); Quantile regression; Stunting; Trend; Socioeconomic; Demographic; LMS method

**Background**

In India, malnutrition has always been a significant public health problem among under-five children. The survival and early development of children have been shown to be severely affected by malnutrition. In addition, it vigorously affects the health of pregnant and nursing mothers. The overall resistance-to diseases and future performance in school and at work was also associated with Malnutrition [1, 2]. In the world, among under-five children, about 3.5 million under-five year old children die each year due to child malnutrition. In developing countries, more than one-third of all child deaths under five were mainly caused by child undernutrition [3]. Therefore, nutrition was a significant factor in the determination of health sector, especially in developing countries [4].

The term malnutrition includes undernutrition and overnutrition, and a significant causative factor for child mortality worldwide [5]. In the realm of child nutrition, the three broadly known pointers to describe the physical growth of children were stunting (short height for age), wasting (low weight for height/length) and underweight (low weight for age) [6-8]. Among the children aged 0-59 months, there was a 38%, 36%, and 21% prevalence of stunted children, underweight, and wasting respectively, according to National Family Health Survey (NFHS-4) [9]. Globally, 165 million children under five years of age were affected by stunting [10]. Stunting and other forms of undernutrition reduce a child's chance of survival. It was characterized as the percentage of children aged 0 to 59 months whose height for age was below minus two standard deviations (moderate stunting) and minus three standard deviations (severe stunting) from the median of the WHO Child Growth Standards [11,12]. To measure child growth, the height for age Z-score (HAZ) was used [may need reference].

In early childhood, particularly stunting increases the chance of susceptibility to sickness. Since stunting was associated with suboptimal brain development, it has practically permanent effects on physical, mental development and was probably going to have long- lasting adverse ramifications for intellectual capacity, school performance, and future benefits [10]. The effects mentioned above on development and uniformity caused by child stunting were most likely to have the outcome of keeping children at the midpoint of poverty, which in turn adversely affects the development of nations [13, 14]. Thus, improving children's nutritional status and lessening the prevalence of malnutrition to advance their physical and mental improvement would be extraordinarily significant [15]. Better wellbeing and nourishment have resulted in financial development just as value concerns [16, 17]. According to the WHO, stunting was a reliable measure of overall social deprivation [18]. Hence, the World Health Assembly has set a global target to reduce the number of stunted under-five children to 40 percent by 2025 [10, 13].

Child health has been predominantly noteworthy due to its connection to child poverty and the build-up of adult human capital. The improvement of needy children's health and nutrition has been an effective method of improving school participation and upgrading financial development since learning converts into gains in long-run productivity [19,20].

There has been considerable literature analysing the determinants of nourishing child status in a broad scope of nations and utilizing distinctive econometric techniques. The studies that were conducted earlier have used standard multiple linear or logistic regression models. The more significant part of those past investigations was that they concentrated on finding the determinants at the mean and odds using logistic regression [21-27]. The utilization of these methods may prompt inaccurate strategy intercession measures if the relationship between child nutritional status and the specific socioeconomic and demographic determinants is heterogeneous at the various percentiles of the nutritional distribution. Moreover, just a couple of studies focused on the distinction at various points of the conditional nutritional dispersion [23,27,28].

The limitation of previous studies can be resolved by analysing demographic and socioeconomic factors across varied points of the conditional stunting (HAZ) distribution in India. Moreover, the changes in the stunting distribution of children and connections between the key covariates patterns and trends in stunting were investigated by using the quantile regression model [29]. << perhaps a note on why you are not using the HAD model >>

The objective of this paper was to find the association between demographic, socioeconomic and health factors of child nutritional status from 4 rounds of National Family Health Survey (NFHS) data of children under 3 years of age years in India over 24 years by using quantile regression. In our opinion, adequate research has not been adequate in this area. The current analysis expects to improve the structure of successful intercession measures designed to tackle child malnutrition and improve child health.

**Methods**

**Data**

Data for this analysis were taken from four consecutive rounds of the NFHS of India conducted during 1992–93, 1998–99, 2005–06, and 2015-2016. The NFHS is a large-scale community-based survey, carried out at household level, in the states and the Union territories of India. The International Institute for Population Sciences (IIPS), Mumbai, India, directed the numerous rounds of the survey with community-oriented assistance from a few national and international associations [30]. The surveys conducted led to more information on reliable estimates of fertility, Infant and children mortality, nutritional status of children, better use of MCH services at a national, state-level and across the urban and rural residence. The initial three rounds of the NFHS were intended to give state-level information. Nonetheless, the fourth round of study, which includes a more prominent sample size, yields assessments of most factors for all 640 districts in India [9]. Each of the four rounds of the study incorporated multi-stage sampling design, two-stage sampling design in rural areas, and three stages in urban areas [31]. The NFHS gathered information utilizing distinctive interview schedules, household schedules and qualified women/individual schedules, and for the fourth round, the Biomarker Questionnaire was incorporated [nine]. In all the rounds, the substance of the schedule remains the same.

The household response rate of the first round was 96% and it was 98 % for the remaining three surveys. The individual response rate for the first, and second, third and fourth rounds was 96%, 96%, 94% and 97%, respectively. Rounds were 96%, 94% for the third round, while 97% in the fourth round of the survey. All four rounds of the NFHS furnish data on anthropometric pointers with a different age group of children. For instance, NFHS-1 gathered data from children aged below four years and NFHS-2 gathered data from children below three years, while NFHS-3 and 4 gathered data from children below five years. Hence, to make the assessments equivalent, the examination was limited to children aged indicators of unit-level data of 206579 children aged <3 years (N=106136 male and 100443 female) over 24 years period were considered.

**Dependent variable**

In this analysis, only HAZ as a dependent variable to find the independent variable’s distribution on different quantiles was used. To estimate the HAZ marker, the reference population of the WHO 2006 was considered [32]. As per the WHO, the child was classified as stunted if the height-for-age Z-score of a child is < - 2 standard deviations (SDs), then the child was classified as stunted. If the child's Z score value is < - 3, it was classified as severely stunted. The HAZ score value considered from -6 to +6 remaining cases were excluded/flagged.

**Independent variable**

Five quantiles of the socioeconomic variable wealth index have been taken into account in this study and the quantiles are (poorest, poorer, middle, richer, and richest). Demographic variables such as caste - Scheduled Caste (SC), Scheduled Tribe (ST), Other Backward Class (OBC), and Other castes were taken into consideration. Child characteristics such as child’s age in months (0-6, 7-12, 13-24 and 25-59), birth order (1, 2, 3, 4 and above), and size at the

time of birth (small, average and large) were taken into consideration. The maternal factors include maternal age of 15-49 years, BMI of mothers (underweight: Body mass index <18.5, normal: 18.5<BMI<25, and overweight BMI>25.0), and mother's education (No education, <5 years of schooling, 5-7, 8-9, 10-11, 12 years or more) were considered. Since wealth index data was not present in the NFHS 1 and 2 surveys, the wealth index was estimated only for the NFHS 3 and 4 surveys. Furthermore, the BMI (maternal) predictor data was not present in the NFHS1 survey; however, it was evaluated for the NFHS 2, 3, and 4 surveys.

**Statistical Analysis**

**Quantile Regression**

Koenker and Bassett (1978) first introduced the key idea of quantile regression [13, 33, 34, 35]. This procedure has an advantage over the conventional common least-squares method. It does not accept a steady effect of the independent factors over the dependent variable's whole distribution [29, 33]. This methodology was utilized to consider a heterogeneous impact of every determinant alongside various percentiles of the dependent variable's conditional distribution [31].

Koenker and Bassett (1978) show that the empirical quantile function is the solution of the minimization problem defined by [33]:

With	can be defined as:

Let	where i= 1, …, n a sample, a	vector of regressors,	,	,
 is the check function, and I(•) usual indicator function.

In this study, quantile regression analysis was performed to identify the independent variables related to child anthropometric Z score over the seven (5^th^, 10^th^, 25^th^, 50^th^, 75^th^, 90^th^, and 95th) percentiles. This analysis was performed using the SAS University Edition software.

**Lambda Mu Sigma (LMS) Method**

Using quantile regression, this study investigated longitudinal changes in the HAZ distribution overtime for the separate sex-stratified. In addition, we used the LMS method to

determine the age-specific secular trends in the child nutritional status measures, thereby allowing us to examine the temporal trends in selected seven percentile points of the stunting.

The LMS method summarizes the shifting distribution by three curves representing the median, coefficient of variation and skewness; the latter expressed as a Box-Cox power [36]. For a given value of covariate, to transform the response to standard normality LMS technique applies a Box-Cox transformation. In order to get the quantiles, an opposite Box- Cox transformation was applied to the quantiles of the standard normal distribution [37, 38]. A large sample size is required to gauge the percentiles in each age group with sufficient accuracy. The division may lose data from nearby groups. To maintain a strategic distance from division, Cole and Green (1992) proposed a Box-Cox transformation based on the semiparametric method from the LMS technique presented by Cole in 1988[39].

We utilised the LMS technique in the GAMLSS package R version 3.4.3 (R Development Core Team, Vienna, Austria) to obtain figures of stunting distribution for 1992 and 2016, along with the lines showing the adjustments in the stunting measures [40, 41]. Additionally, we utilised the LMS method to decide the age-specific patterns in the HAZ measures for both girls and boys, along with the lines permitting us to analyse the worldly patterns in explicit percentile purposes of the HAZ.

**Results**

Table 1 presents descriptive statistics for the individual demographic, socioeconomic and health-related variables for each sex.

The calculated coefficients for seven percentiles (5th, 10th, 25th, 50th, 75th, 90th, and 95th) of males and females were calculated separately for the NFHS 1, 2, 3 and 4 are presented in Tables 2 through 5.

From the quantile regression results, it is observed that child age in months for both girls and boys for the categories (25-36, 13-24, 7-12) was negatively associated and was statistically significant across the quantiles. If the child's age increases, the coefficient values increase and these values are low in the lower quantiles compared to the upper quantiles. When the age and quantiles increase the coefficient, values increase compared to the reference age group (0-6 months), it indicates that in the first six months of life, HAZ were better and when they grow older, the children become vulnerable to malnutrition. Although these values hold for all surveys, boys were found to have better HAZ values than the girls did across the quantiles; however, the coefficient values' strength is different in various quantiles.

The factor children's mother's education, while comparing the reference category (>12 years of education) with (No education, <5, 5-7, 8-9, 10-11 years) showed a negative association and statistically significant association in percentiles for the boys and girls, although girls whose mother having the education of 8-9 and 10-11-years education showed a positive association observed in Table 3 and 4 for the 95th percentile. It was also observed that the children's HAZ coefficient values decreased when their mother's education increased from lower education to higher education. It indicates that, as the mothers get more educated, their children get less vulnerable to malnutrition. Educated mothers have been shown to prioritise meeting the nutritional requirements of the children. They tend to analyse the prevailing childcare practices as well as the available health facilities within their reach. Therefore, children of better-educated mothers have been shown to be well looked after as compared to ‘not so well educated’ mothers.

A child's size at birth (for category: small and average) was found to be statistically significant and be negatively associated with HAZ scores across quantiles in the first, second, and fourth rounds of the survey. Suppose one moves from lower percentile to upper percentile, the value of HAZ increases. For the NFHS 3 child size at birth (category: average) was negatively associated with the reference category (Size at birth: Large). It indicates that the size at birth plays a main role in the area of child nutrition. Also observed that girls are having better HAZ outcomes when compared to boys. The size of a newborn baby at birth plays a significant role in malnutrition. This analysis showed that children are larger size at birth are less likely to be vulnerable to malnutrition as compared to the children who were smaller and of average size.

In all the rounds, the caste factor type had a significant role. Children whose caste belonged to the SC category had a negative and statistically significant result compared to those children belonging to other castes. In the third round, among OBC category of castes, male children had a negative statistically significance (from 10^th^ to 90^th^ percentile) associated with the lower quantiles, and it was positively associated at 95th percentile. In fourth round, the type of caste (children who belong to OBC) in male children had a negative statistically significant association (except 10^th^ percentile).

In all, the rounds of NFHS survey Birth order (category: four or more, 3) was found have a negative association and statistically significant in the lower tails than the upper tails of the distribution compared with the reference category. It also revealed that children were more prone to be nutritionally vulnerable when the birth order increased from two to four. Mothers, nutritional status (category: BMI<18.5) showed negatively significant association in the second, third and fourth round of NFHS and these coefficient values are low in the lower tails compared to the upper tails of the distribution. It indicates that children whose mothers BMI <18.5 are more likely to be stunted than their best peers are. Children whose mothers BMI (>25) had a positive association in all percentiles compared to the reference category. It reveals that the higher BMI values of mother's children are less likely to stunted when compared with children's mother's nutritional status is normal, i.e., BMI (18.5-25). In this study, the wealth index was calculated for the three and fourth rounds of the survey due to no data available for the first and second rounds of the survey. The wealth index for both sexes for the categories (categories: poorest, poorer, middle, richer) was negatively and statistically significant with the whole percentile of the distribution. If the child belonging to the wealth status was the poorest, the coefficient values were high in lower tails and these values were low in the higher quantiles compared to the reference group.

The economic condition of the family often influences the deprivation of infants. In this analysis, we find that among children with the lowest socioeconomic history the probabilities for being stunted, underweight and wasted were substantially greater. As those families with lower incomes have a minimal supply of food, they cannot ensure appropriate food security and thus their growth and development get affected. Health care cannot meet their essential needs, influencing development and growth.

Figure 1 presents the smoothed distribution curve using the LMS method for the HAZ differences over time in males and females aged 0-36 months in selected years. A decreasing trend is seen in all of the percentile curves from 1992 to 2016, in which the levels are decreased in the higher percentile levels among both sexes and in each age group. Differentiation of the plots noted that females' stunting percentile curves were marginally stable than those of male children. For girls and boys, as age increases, the results indicate a higher propensity for stunting. However, when compared to the NFHS 1 with NFHS, the four curves were less decreased.

**Discussion**

Using the data obtained over a long period of 24 years from the four rounds of NFHS, the determinants associated with HAZ and trend in the HAZ distribution among children aged 0-36 months of data using quantile regression were identified. The LMS method was used to construct the curves of the 5^th^, 10^th^, 25^th^, 50^th^, 75^th^, 90^th^, and 95^th^ percentiles. There have been research studies carried out worldwide that investigated the determinants of child nutritional status in a wide scope of nations and utilizing distinctive econometric techniques. Most of the past investigations have generally utilized standard linear regression and logistic regressions.

The utilization of these regression estimation techniques may prompt the misdirection of policy intervention measures, if the relationship between child nutritional status and the different determinants are heterogeneous at the various percentiles [42]. One of the most important objectives in the agenda of the developing countries is to decrease child malnutrition. This paper embodies a quantile regression approach to investigate the effects of demographic, socio-economic and health-related factors on HAZ at different percentiles [13]. The analysis identified the child's age in months as one of the strongest determinants of child nutritional status. Results showed a negative increment in coefficients from lower percentiles to upper percentiles than the first six months of life. The analysis also reveals stunting in the first six months of life, and is increases with age [23, 43, 44].

The present study showed a negative association between the mother's educational status and HAZ. Children of mothers with no education, 12 years. It was observed that the children of mothers with less education had worse HAZ scores than children of mothers with high education. This analysis provides evidence that a mother's educational status plays a crucial role in child nutrition. Educational status, especially of mothers, has been shown to accomplishments significantly influence the Knowledge-Attitude-Practices pertaining to nutrition as well as the risks associated with inadequate food intake of nutritional information, awareness and risks associated with insufficient food. Well-educated mothers were capable enough to feed their children more satisfactorily owing to their better educational status. They were shown to utilise the available health facilities in more optimum ways and their health-related behaviours, including nutrition related knowledge and practices were found to be quite satisfactory. and nutrition-rich foods [45-47].

The present study showed a negative association and statistical significance between household economic status and HAZ. Children from the poorest, poorer, and middle household economic status had higher negative HAZ scores than the richest households. It is observed that the children of low-income families had worse HAZ scores than children from high-income households. The study illustrates that factors like child age, birth order of the child, mother's education and household economic status were more important than aggregate economic conditions [48]. For the factor ‘size at birth’, it was found that those male children whose weight at birth was less than desired suffered a nutritional drawback in height as compared to their best peers.

The mother's nutritional status (BMI) was also associated and statistical significance with children's nutrition. The results showed a substantial effect on a mother's BMI on a child’s nutritional status. Children of mothers with lower BMI are more likely to be less HAZ when compared with their counterparts.

**Strength and limitations of the study**

The study showed trends in child nutritional status from 1992 to 2016 using the four rounds of NFHS data covering most of India. A more effective approach using quantile regression and LMS method to identify the heterogeneous effect of the child’s nutritional status with household, socio-economic and health-related determinants.

Various intervention measures are required to consider the different effects of child’s nutritional status determinants with various percentiles of the HAZ distribution. Child malnutrition can only be tackled with a multifaceted approach that includes targeted intervention, and it cannot be combated with a one-size-fits-all policy [42].

LMS curve with different percentiles for 1992 and 2016 quantified the trends in the performance of each sex concerning child’s nutritional status. This study investigates how factors associated with a child's nutritional status affect the stunting distribution on the selected percentiles. Limitations of the analysis based on secondary data also apply to this study. Cross-section data was used for this study.

**Conclusion**

This quantile regression suggests that the effects of different covariates worked differently across the HAZ distribution. Predictors of stunting viz: child’s age in months, birth order, size at birth, and wealth index and their associations vary across different quantiles of stunting in India. Since India's socio-economic and demographic characteristics have underlined the significant disparities in many aspects, national strategies to tackle stunting should be tailored appropriately for various segments. It would be adequate to carefully integrate applicable interventions according to the objective and target population for individuals’ wellbeing and the country's development. Various measures could effectively combat child nutrition among lower socio-economic strata: Increased awareness among family members on the right feeding practices using mass media or other community-based programs, subsidizing the cost of nutrition-rich foods and ensuring adequate universal health care coverage.

**Funding:** Not applicable

**Availability of data and materials**

The secondary data "NFHS" used in this study are publicly available at <https://dhsprogram.com/what-we-do/survey/survey-display-355.cfm>

**Authors' contributions**

Thirupathi Reddy M contributed to the data collection, analysis, and manuscript preparation. Vishnu Vardhana Rao M developed the study protocol, secured funds, supervised the study, and guided in manuscript preparation.

**Ethics approval and consent to participate**

This study is based on a publicly accessible survey dataset; therefore, this request is not applicable.

**Competing interests**

The authors declare that they have no competing interests.

**Acknowledgements:** The authors gratefully acknowledge the financial support and fellowship granted by the National Institute of Nutrition (NIN), Indian Council of Medical Research, Hyderabad, India, for carrying out the study. The authors wish to thank Dr. R Hemalatha (Director, NIN) for her encouragement. The authors would also like to thank Dr. A. Laxmaiah Scientist G & HOD, Public Health Nutrition Division, NIN.

**Table 1** Descriptive Statistics and prevalence of stunting children aged 0-36 months by factors at different categories based on (NFHS- 1, 2, 3, 4) data.

**Variable (n and %) in each category)**

|  | Category | **NFHS-1** | | **NFHS-2** | | **NFHS-3** | | **NFHS-4** | |
| --- | --- | --- | --- | --- | --- | --- | --- | --- | --- |
|  |  | **Female** | **Male** | **Female** | **Male** | **Female** | **Male** | **Female** | **Male** |
| **Child age in months**  **Type of caste**  **Mothers Education**  **Size at birth**  **Birth order**  **Wealth Index**  **Mothers BMI** | 25-36  Missing  SC  Missing  No education  8-9 years  Missing  (Small)  Missing  4 or more  Missing  Poorest  Richer  Missing  <18.5  Missing | 3175(29.8%) 3343(30.6%)  0(0%) 0(0%)  1313(12.3%) 1445(13.2%)  0(0%) 0(0%)  5927(55.6%) 6143(56.1%)  938(8.8%) 1068(9.8%)  39(0.37%) 39(0.4%)  2369(22.2%) 2129(19.5%)  100(0.9%) 71(0.6%)  3130(29.4%) 3270(29.9%)  0(0%) 0(0%)  - -  - -  - -  - -  - - | | 3581(30.3%) 3934(30.5%)  0(0%) 0(0%)  2156(18.2%) 2382(18.5)  82(0.7%) 63(0.5%)  5784(48.9%) 6140(47.7%)  1210(10.2%) 1486(11.5%)  6(0.05%) 5(0.04%)  3144(26.6%) 2970(23.1%)  8(0.07%) 6(0.05%)  3090(26.1) 3395(26.3%)  0(0%) 0(0%)  - -  - -  - -  4152(35.1%) 4661(36.2%)  50(0.4%) 69(0.5%) | | 4041(33.5%) 4456(34.5%)  0(0%) 0(0%)  2080(17.3%) 2292(17.7%)  512(4.3%) 572(4.4%)  4731(39.3%) 4916(38.1%)  1812(15%) 1965(15.2%)  0(0%) 0(0%)  2668(22.1%) 2511(19.4%)  168(1.4%) 170(1.3%)  2712(22.5%) 2893(22.4%)  0(0%) 0(0%)  2165(18%) 2171(16.8%)  2689(22.3%) 2948(22.8%)  0(0%) 0(0%)  3970(32.9%) 4276(33%)  80(0.7%) 61(0.5%) | | 22001(33.4%) 23430(33.3%)  0(0%) 0(0%)  12527(19%) 13201(18.8%)  2979(4.5%) 3043(4.3%)  19260(29.2%) 20041(28.5%)  12082(18.3%) 13279(18.9%)  0(0%) 0(0%)  8333(12.6%) 7985(11.3%)  1287(2%) 1264(1.8%)  10122(15.4%) 10800(15.3%)  0(0%) 0(0%)  16886(25.6%) 17645(25.1%)  11254(17.1%) 11886(16.9%)  0(0%) 0(0%)  16004(24.3%) 17596(25%)  413(0.6%) 405(0.6%) | |

13-24	3637(34.1%)	3730(34.1%)	3948(33.4%)	4222(32.8%)	3945(32.7%)	4302(33.3%)	21911(33.2%)	23273(33.0%)

7-12	1663(15.6%)	1715(15.7%)	1925(16.3%)	2118(16.4%)	2019(16.8%)	2150(16.6%)	10881(16.5%)	12007(17.1%)

0-6	2181(20.5%)	2153(19.7%)	2373(20.1%)	2611(20.3%)	2048(17%)	2008(15.5%)	11114(16.9%)	11684(16.6%)

ST	1268(11.9%)	1262(11.5%)	1783(15.1%)	1822(14.1%)	2018(16.7%)	1935(15%)	13292(20.2%)	13493(19.2%)

OBC	-	-	3419(28.9%)	3660(28.4%)	3930(32.6%)	4262(33%)	25747(39.1%)	28179(40%)

Other caste	8075(75.8%)	8234(75.3%)	4387(37.1%)	4958(38.5%)	3513(29.1%)	3855(29.8%)	11362(17.2%)	12478(17.7%)

<5 years	739(6.9%)	703(6.4%)	1071(9.1%)	1090(8.5%)	867(7.2%)	911(7.1%)	4062(6.2%)	4111(5.8%)

5-7 years	1462(13.7%)	1409(12.9%)	1831(15.5%)	1856(14.4%)	1932(16%)	1946(15.1%)	10321(15.7%)	11294(16%)

10-11 years	848(8.0%)	862(7.9%)	997(8.4%)	1185(9.2%)	1095(9.1%)	1383(10.7%)	7638(11.6%)	8075(11.5%)

>12 years	703(6.6%)	71(6.6%)	928(7.8%)	1123(8.7%)	1616(13.4%)	1795(13.9%)	12544(19%)	13594(19.3%)

Average	6921(64.9%)	7177(65.6%)	7071(59.8%)	7858(61%)	6612(54.9%)	7214(55.9%)	45296(68.7%)	48796(69.3%)

Large	1266(11.9%)	1564(14.3%)	1604(13.6%)	2051(15.9%)	2605(21.6%)	3021(23.4%)	10991(16.7%)	12349(17.5%)

Three	1918(18%)	1951(17.8%)	2061(17.4%)	2286(17.7%)	1911(15.9%)	2147(16.6%)	10127(15.4%)	11487(16.3%)

Second	2669(25%)	2668(24.4%)	3126(26.4%)	3467(26.9%)	3445(28.6%)	3762(29.1)	20912(31.7%)	22162(31.5%)

First	2939(27.6%)	3052(27.9%)	3550(30%)	3737(29%)	3985(33.1%)	4114(31.9%)	24746(37.5%)	25945(36.9%)

Poorer	-	-	-	-	2288(19%)	2256(17.5%)	15704(23.8%)	16535(23.5%)

Middle	-	-	-	-	2487(20.6%)	2692(20.8%)	13318(20.2%)	14374(20.4%)

Richest	-	-	-	-	2424(20.1%)	2849(22.1%)	8745(13.3%)	9954(14.1%)

BMI>25	-	-	668(5.6%)	699(5.4%)	1044(8.7%)	1208(9.4%)	8377(12.7%)	8972(12.7%)

BMI 18.5-25	-	-	6957(58.8%)	7456(57.9%)	6959(57.7)	7371(57%)	41113(62.4%)	43421(61.7%)

**-** : No Data available , ( )	percentage of data

**Table 2:** Quantile Regression Estimations for the different quantiles of the female (10517) and male (10830) children's, Dependent variable (HAZ) NFHS-1

|  | **0.05** | | **0.1** | | **0.25** | | **0.5** | | **0.75** | | **0.9** | | **0.95** | |
| --- | --- | --- | --- | --- | --- | --- | --- | --- | --- | --- | --- | --- | --- | --- |
| Parameter | Female | Male | Female | Male | Female | Male | Female | Male | Female | Male | Female | Male | Female | Male |
| Child age in Months  25-36in Months  13-24 months  7-12 months  0-6 months  Mothers Education  No education  <5 years  5-7 years  8-9 years  10-11 years  >12 years  Size at birth  Small  Average  Large  Type of caste  (SC)  ST  Other caste  Birth order  4 or more  Three  Second  First | -0.97* -0.63*  -0.68* -0.52*  -0.32* -0.25**  Reference Reference    -0.68* -0.73*  -0.33* -0.21  -0.14 -0.27***  -0.02 -0.03  -0.05 0.09  Reference Reference      -0.35* -0.21***  -0.22** -0.04  Reference Reference      -0.05 -0.06  -0.33* -0.21***  Reference Reference      -0.13 -0.19**  -0.07 -0.03  -0.03 0.02  Reference Reference | | -1.38* -0.94*  -1.01* -0.80*  -0.50* -0.33*  Reference Reference    -1.03* -1.13*  -0.55* -0.49*  -0.35* -0.60*  -0.36* -0.26***  -0.006 -0.05  Reference Reference      -0.34* -0.50*  -0.24* -0.23*  Reference Reference      -0.11 -0.17**  -0.12 -0.16***  Reference Reference      -0.14** -0.12  -0.03 -0.06  0.01 0.04  Reference Reference | | -1.7* -1.28*  -1.32* -1.08*  -0.56* -0.40*  Reference Reference    -1.21* -1.26*  -0.82* -0.77*  -0.59* -0.70*  -0.45* -0.49*  -0.23 ** -0.25  Reference Reference      -0.45* -0.48*  -0.23* -0.17*  Reference Reference      -0.15** -0.18*  0.12*** -0.07  Reference Reference      -0.15** -0.08  0.04 0.004  0.05 0.04  Reference Reference | | -1.75* -1.56*  -1.40* -1.35*  -0.64* -0.50*  Reference Reference    -1.05* -1.11*  -0.81* -0.80*  -0.61* -0.62*  -0.47* -0.44*  -0.16*** -0.25*  Reference Reference      -0.53* -0.58*  -0.29* -0.28*  Reference Reference      -0.13** -0.15*  0.17* 0.09***  Reference Reference      -0.13* -0.08***  -0.02 -0.003  0 0.01  Reference Reference | | -1.80* -1.8*  -1.40* -1.52*  -0.71* -0.67*  Reference Reference    -0.76* -1.09*  -0.67* -0.77*  -0.44* -0.73*  -0.29* -0.49*  -0.02 -0.29*  Reference Reference      -0.57* -0.63*  -0.32* -0.28*  Reference Reference      -0.23* -0.12***  0.24* 0.31*  Reference Reference      -0.13** -0.04  -0.11*** -0.08  -0.02 0.04  Reference Reference | | -1.89* -1.93*  -1.55* -1.63*  -0.79* -0.78*  Reference Reference    -0.74* -0.82*  -0.70* -0.70*  -0.60* -0.57*  -0.55* -0.67*  -0.21 -0.33 **  Reference Reference      -0.69* -0.62*  -0.39* -0.31*  Reference Reference      -0.18** -0.06  0.53* 0.58*  Reference Reference      -0.001 -0.11  -0.11 -0.1  -0.01 0.06  Reference Reference | | -2.00* -1.80*  -1.57* -1.48*  -0.95* -0.67*  Reference Reference    -0.46** -0.59*  -0.60** -0.48  -0.43** -0.39  -0.68* -0.63**  0.25 -0.17  Reference Reference      -0.94* -0.69*  -0.39** -0.39**  Reference Reference      -0.17 -0.13  0.93* 1.03*  Reference Reference      -0.09 -0.15  -0.33** -0.04  -0.11 0.1  Reference Reference | |

*: 1% Significance; **: 5% Significance; ***: 10% Significance

**Table 3**: Quantile Regression Estimations for the different quantiles of the female (11582) and Male (12742) children’s, Dependent variable (HAZ) NFHS-2

| Parameter | 0.05 | | 0.1 | | 0.25 | | 0. 50 | | 0.75 | | 0.9 | | 0.95 | |
| --- | --- | --- | --- | --- | --- | --- | --- | --- | --- | --- | --- | --- | --- | --- |
|  | Female | Male | Female | Male | Female | Male | Female | Male | Female | Male | Female | Male | Female | Male |
| Child age  25-36in Months  13-24 months  7-12 months  0-6 months  Mothers Education  No education  <5 years  5-7 years  8-9 years  10-11 years  >12 years  Size at birth  Small  Average  Large  Type of cast  SC  ST  OBC  Other caste  Birth order  4 or more  Three  Second  First  Mothers BMI  BMI<18.5  BMI>25  BMI 18.5-25 | -1.75* -1.32*  -1.52* -1.20*  -0.48* -0.54*  Reference Reference    -1.22* -1.15*  -0.95* -0.79*  -0.52* -0.73*  -0.64* -0.52*  -0.50* -0.46*  Reference Reference      -0.42* -0.53*  -0.16*** -0.27*  Reference Reference      -0.21* -0.18**  -0.06 -0.18***  -0.07 -0.22*  Reference Reference    -0.29* -0.22*  -0.12 -0.04  -0.1 -0.0575  Reference Reference  -0.01 -0.07  0.40* 0.2  Reference Reference | | -1.81* -1.48*  -1.45* -1.33*  -0.51* -0.59*  Reference Reference    -1.21* -1.18*  -0.87* -0.74*  -0.54* -0.81*  -0.52* -0.49*  -0.28** -0.35*  Reference Reference      -0.45* -0.56*  -0.13*** -0.33*  Reference Reference      -0.16** -0.27*  0.01 -0.25*  -0.14** -0.25*  Reference Reference    -0.31* -0.25*  -0.08 -0.11***  -0.1 -0.067  Reference Reference  -0.05 -0.07  0.37* 0.27*  Reference Reference | | -1.75* -1.51*  -1.41* -1.41*  -0.62* -0.61*  Reference Reference    -1.11* -1.20*  -0.69* -0.81*  -0.55* -0.71*  -0.40* -0.49*  -0.30* -0.35*  Reference Reference      -0.55* -0.57*  -0.24* -0.34*  Reference Reference      -0.19* -0.23*  0.12** -0.14**  -0.05 -0.11**  Reference Reference    -0.33* -0.23*  -0.13** -0.08  -0.08*** -0.04  Reference Reference  -0.08** -0.09*  0.51* 0.29*  Reference Reference | | -1.87* -1.63*  -1.51* -1.53*  -0.69* -0.67*  Reference Reference    -1.04* -0.98*  -0.69* -0.65*  -0.57* -0.67*  -0.48* -0.37*  -0.33* -0.25*  Reference Reference      -0.60* -0.59*  -0.28* -0.32*  Reference Reference      -0.19* -0.29*  0.056 -0.13*  -0.05 -0.08***  Reference Reference    -0.29* -0.23*  -0.18* -0.09***  -0.11** -0.06  Reference Reference  -0.09* -0.17*  0.428* 0.24*  Reference Reference | | -2.02* -1.89*  -1.72* -1.64*  -0.84* -0.78*  Reference Reference    -0.82* -0.86*  -0.51* -0.64*  -0.50* -0.55*  -0.33* -0.33*  -0.17*** -0.2  Reference Reference      -0.52* -0.56*  -0.22* -0.32*  Reference Reference      -0.18* -0.21*  0.15** -0.04***  -0.09*** -0.04  Reference Reference    -0.17* -0.13  -0.61 -0.11***  -0.07 -0.05  Reference Reference  -0.17* -0.24*  0.40* 0.28**  Reference Reference | | -2.20* -2.24*  -1.87* -1.95*  -1.01* -1.03*  Reference Reference    -0.66* -0.76*  -0.43* -0.77*  -0.41* -0.60*  -0.30** -0.42*  -0.15 -0.19  Reference Reference      -0.50* -0.61*  -0.26* -0.42*  Reference Reference      -0.20* -0.26*  0.16** 0.14***  -0.13*** -0.07  Reference Reference    0.03 0.05  -0.02 -0.14***  0.03 -0.03  Reference Reference  -0.28* -0.28*  0.36* 0.28**  Reference Reference | | -2.17* -2.28*  -1.93* -1.93*  -1.01* -0.96*  Reference Reference    -0.38** -0.61*  -0.31 -0.55*  -0.23 -0.57*  -0.1 -0.42**  -0.1 -0.30***  Reference Reference    -0.49* -0.55*  -0.23*** -0.36*  Reference Reference    -0.27** -0.26**  0.30** 0.43*  -0.20*** -0.03  Reference Reference    0.29** 0.17  -0.06 -0.20***  -0.03 0.08  Reference Reference  -0.44* -0.50*  0.19 0.23  Reference Reference | |

*: 1% Significance; **: 5% Significance; ***: 10% Significance

**Table 4:** Quantile Regression Estimations for the different quantiles of the female (11315) and male (12133) children's, Dependent variable (HAZ) NFHS-3

| **Parameter** | **0.05** | | **0.1** | | **0.25** | | **0.5** | | **0.75** | | **0.9** | | **0.95** | |
| --- | --- | --- | --- | --- | --- | --- | --- | --- | --- | --- | --- | --- | --- | --- |
|  | Female | Male | Female | Male | Female | Male | Female | Male | Female | Male | Female | Male | Female | Male |
| Child age in months  25-36in Months  13-24 months  7-12 months  0-6 months  Mothers Education  No education  <5 years  5-7 years  8-9 years  10-11 years  >12 years  Size at birth  (Small)  Average  Large  Type of caste  SC  ST  OBC  Other caste  Birth order  (4 or more)  Three  Second  First  Mothers nutritional status  BMI<18.5  BMI>25  BMI 18.5-25  Wealth Index  (Poorest)  Poorer  Middle  Richer  Richest | -0.86* -0.89*  -0.86* -0.87*  -0.16 -0.26**  Reference Reference      -0.79* -0.50*  -0.90* -0.19  -0.31** -0.30**  -0.31** -0.15  -0.12 -0.21***  Reference Reference    -0.32* -0.31*  -0.11 -0.03  Reference Reference    -0.1 -0.11  0.04 -0.04  -0.14*** -0.1  Reference Reference  -0.25* -0.51*  -0.15 -0.24*  -0.04 -0.22*  Reference Reference    -0.07 -0.14**  0.11 0.08  Reference Reference    -0.73* -0.72*  -0.52* -0.73*  -0.40* -0.50*  -0.46* -0.26**  Reference Reference | | -1.05* -1.02*  -0.98* -1.01*  -0.35* -0.41*  Reference Reference      -0.64* -0.55*  -0.59* -0.14  -0.24* -0.36 *  -0.21** -0.14  0.05 -0.13  Reference Reference    -0.35* -0.39*  -0.10*** -0.03  Reference Reference    -0.16** -0.23*  0.01 -0.09  -0.14* -0.17*  Reference Reference  -0.29* -0.37*  -0.18* -0.17**  -0.08 -0.07  Reference Reference    -0.08*** -0.14**  0.16** 0.07  Reference Reference    -0.79* -0.82*  -0.52* -0.71*  -0.49* -0.53*  -0.40* -0.30*  Reference Reference | | -1.36* -1.28*  -1.26* -1.28*  -0.46* -0.56*  Reference Reference      -0.51* -0.58*  -0.47* -0.28*  -0.24* -0.40*  -0.18** -0.24*  -0.07 -0.15***  Reference Reference    -0.35* -0.40*  -0.12* -0.10**  Reference Reference    -0.24* -0.24*  0.05 -0.06  -0.13* -0.15*  Reference Reference  -0.25* -0.25*  -0.11** -0.16*  -0.11** -0.05  Reference Reference    -0.16* -0.17*  0.18* 0.09  Reference Reference    -0.73* -0.80*  -0.52* -0.60*  -0.42* -0.48*  -0.26* -0.30*  Reference Reference | | -1.51* -1.47*  -1.42* -1.45*  -0.54* -0.63*  Reference Reference      -0.55* -0.63*  -0.58* -0.37*  -0.32* -0.50*  -0.24* -0.39*  -0.17* -0.33*  Reference Reference    -0.27* -0.45*  -0.06 -0.15*  Reference Reference    -0.24* -0.23*  0.09*** -0.04  -0.12* -0.15*  Reference Reference  -0.18* -0.18*  -0.05 -0.15*  -0.04 -0.09**  Reference Reference    -0.21* -0.18*  0.16* 0.12**  Reference Reference    -0.64* -0.66*  -0.48* -0.57*  -0.39* -0.31*  -0.24* -0.20*  Reference Reference | | -1.68* -1.67*  -1.58* -1.58*  -0.57* -0.68*  Reference Reference      -0.42* -0.51*  -0.42* -0.31*  -0.30* -0.41*  -0.21* -0.34*  -0.22* -0.27*  Reference Reference    -0.19* -0.44*  -0.03 -0.19*  Reference Reference    -0.25* -0.23*  0.16* -0.07  -0.12** -0.14*  Reference Reference  -0.16 * -0.08  -0.03 -0.12**  -0.03 -0.01  Reference Reference    -0.18* -0.18*  0.25* 0.11  Reference Reference    -0.64* -0.61*  -0.46* -0.50*  -0.38* -0.37*  -0.25* -0.22*  Reference Reference | | -1.67* -1.72*  -1.59* -1.59*  -0.56* -0.70*  Reference Reference      -0.26** -0.52*  -0.2 -0.26***  -0.29** -0.42*  -0.19 -0.33*  -0.21 -0.29**  Reference Reference    -0.19** -0.42*  -0.08 -0.16**  Reference Reference    -0.09 -0.19**  0.34* 0.14  0.01 -0.05  Reference Reference  -0.07 0.14***  0.04 -0.05  0.02 0.04  Reference Reference    -0.29* -0.18  0.27** 0.01  Reference Reference    -0.54* -0.53*  -0.44* -0.47*  -0.31* -0.44*  -0.16 -0.22**  Reference Reference | | -1.88* -1.56*  -1.74* -1.41*  -0.61* -0.46*  Reference Reference    -0.04 -0.57*  -0.03 -0.12  -0.16 -0.39**  0.06 -0.11  0.07 -0.33**  Reference Reference    -0.13 -0.49*  0.01 -0.18***  Reference Reference    0.04 0.01  0.54* 0.41*  0.05 0.12  Reference Reference  0.09 0.34*  0.05 0.23***  0.01 -0.03  Reference Reference    -0.41* -0.24*  0.01 -0.07  Reference Reference    -0.67* -0.46*  -0.47* -0.43*  -0.43** -0.52*  -0.38** -0.40*  Reference Reference | |

*: 1% Significance; **: 5% S ignificance;

***: 10% Significance

**Table 5:** Quantile Regression Estimations for the different quantiles of the Female (61582) and Male (66018) children’s, Dependent variable (HAZ). NFHS-4

| **Parameter** | **0.05** | | **0.1** | | **0.25** | | **0.5** | | **0.75** | | **0.9** | | **0.95** | |
| --- | --- | --- | --- | --- | --- | --- | --- | --- | --- | --- | --- | --- | --- | --- |
|  | Female | Male | Female | Male | Female | Male | Female | Male | Female | Male | Female | Male | Female | Male |
| Child age  25-36 in Months  13-24 months  7-12 months  0-6 months  Mothers Education  No education  <5 years  5-7 years  8-9 years  10-11 years  >12 years  Size at birth  (Small)  Average  Large  Type of cast  (SC)  ST  OBC  Other caste  Birth order  (4 or more)  Three  Second  First  Mothers BMI  BMI<18.5  BMI>25  BMI 18.5-25  Wealth Index  (Poorest)  Poorer  Middle  Richer | -0.50* -0.12*  -0.52* -0.41*  -0.07 -0.11b  Reference Reference      -0.50* -0.46*  -0.34* -0.27*  -0.30* -0.24*  -0.09*** -0.07  -0.01 -0.08  Reference Reference    -0.35* -0.22*  0.02 -0.02  Reference Reference    -0.10** -0.19*  0.01 -0.20*  -0.06 -0.12*  Reference Reference      -0.30* -0.12**  -0.17* -0.10**  -0.08** 0.02  Reference Reference    -0.11* -0.06***  0.28* 0.26*  Reference Reference    -0.62* -0.56*  -0.48* -0.31*  -0.31* -0.20*  -0.20* -0.08 | | -0.67* -0.36*  -0.68* -0.59*  -0.13* -0.11*  Reference Reference      -0.53* -0.46*  -0.36* -0.26*  -0.30* -0.29*  -0.17* -0.12*  -0.10** -0.08***  Reference Reference    -0.36* -0.27*  -0.002 -0.04  Reference Reference    -0.17* -0.24*  -0.01 -0.18*  -0.12* -0.14*  Reference Reference      -0.25* -0.14*  -0.18* -0.11*  -0.10* -0.03  Reference Reference    -0.13* -0.10*  0.24* 0.25*  Reference Reference    -0.67* -0.62*  -0.47* -0.40*  -0.33* -0.17*  -0.18* -0.09** | | -1.02* -0.82*  -1.00* -1.00*  -0.28* -0.29*  Reference Reference      -0.48* -0.47*  -0.37* -0.31*  -0.30* -0.32*  -0.21* -0.17*  -0.11* -0.09*  Reference Reference    -0.36* -0.41*  -0.05** -0.11*  Reference Reference    -0.20* -0.23*  0.001 -0.09*  -0.15* -0.16*  Reference Reference      -0.23* -0.16*  -0.15* -0.10*  -0.07* -0.06*  Reference Reference    -0.16* -0.15*  0.20* 0.19*  Reference Reference    -0.68* -0.63*  -0.46* -0.39*  -0.32* -0.23*  -0.19* -0.10* | | -1.23* -1.15*  -1.15* -1.21*  -0.35* -0.35*  Reference Reference      -0.43* -0.46*  -0.32* -0.35*  -0.31* -0.31*  -0.25* -0.22*  -0.11* -0.12*  Reference Reference    -0.41* -0.45*  -0.08* -0.13*  Reference Reference    -0.24* -0.22*  0.02 -0.07*  -0.16* -0.16*  Reference Reference      -0.21* -0.12*  -0.14* -0.09*  -0.07* -0.04**  Reference Reference    -0.20* -0.19*  0.15* 0.13*  Reference Reference    -0.60* -0.58*  -0.45* -0.38*  -0.32* -0.25*  -0.17* -0.11* | | -1.41* -1.36*  -1.27* -1.28*  -0.38* -0.37*  Reference Reference      -0.34* -0.42*  -0.32* -0.38*  -0.29* -0.33*  -0.24* -0.24*  -0.12* -0.10*  Reference Reference    -0.48* -0.47*  -0.13* -0.17*  Reference Reference    -0.24* -0.22*  0.05*** -0.07*  -0.18* -0.20*  Reference Reference      -0.18* -0.11*  -0.11* -0.07*  -0.05** -0.03  Reference Reference    -0.27* -0.21*  0.09* 0.12*  Reference Reference    -0.57* -0.51*  -0.46* -0.39*  -0.34* -0.24*  -0.20* -0.12* | | -1.42* -1.41*  -1.20* -1.26*  -0.32* -0.30*  Reference Reference      -0.27* -0.31*  -0.26* -0.40*  -0.24* -0.30*  -0.24* -0.27*  -0.10*** -0.07  Reference Reference    -0.61* -0.54*  -0.2* -0.24*  Reference Reference    -0.21* -0.19*  0.14* 0.05  -0.16* -0.22*  Reference Reference      -0.13* -0.02  -0.06 -0.04  -0.08** 0.03  Reference Reference    -0.42* -0.34*  0.02 0.08***  Reference Reference    -0.54* -0.47*  -0.47* -0.42*  -0.38* -0.25*  -0.30* -0.11** | | -1.37* -1.37*  -1.19* -1.15*  -0.27* -0.28*  Reference Reference    -0.15 -0.31*  -0.30** -0.38*  -0.17*** -0.29*  -0.20** -0.30*  -0.08 -0.1  Reference Reference    -0.64* -0.62*  -0.19** -0.29*  Reference Reference    -0.31* -0.13***  0.29* 0.18**  -0.17** -0.23*  Reference Reference    -0.1 0.07  0.03 0.02  -0.01 0.10c  Reference Reference    -0.57* -0.46*  -0.11 0.03  Reference Reference    -0.53* -0.46*  -0.46* -0.39*  -0.34* -0.18**  -0.23** -0.07 | |

Richest	Reference Reference Reference Reference Reference Reference Reference Reference Reference Reference Reference Reference Reference Reference *: 1% Significance; **: 5% Significance; ***: 10% Significance

**Figure 1**: LMS curves for child aged 0-36 months in NFHS 1 (1992) and NFHS 4 (2016)

**References**

1. Sahu SK, Kumar SG, Bhat BV, Premarajan KC, Sarkar S, Roy G, et al. Malnutrition

among under-five children in India and strategies for control. Journal of Natural
Science, Biology and Medicine. 2015. pp. 18–23. doi:10.4103/0976-9668.149072

2. Agency IAE, Sous-emploi A d’Execution des T d’Interet P contre le. Child nutrition in
Senegal. Available: <https://inis.iaea.org/search/search.aspx?orig_q=RN:34028299>

3. Mokalla TR, Mendu VVR. Risk factors and socioeconomic inequalities in
undernutrition among children 0-59 months of age in India. Int J Popul Stud. 2019;5:

14–23. doi:10.18063/ijps.v5i2.1125

4. UNDP 2002. Introduction The Issues of Public Health and Medical Systems in

Developing Countries. 2002; 1–7.

5. Markos Z. Predicting Under Nutrition Status of Under-Five Children Using Data

Mining Techniques: The Case of 2011 Ethiopian Demographic and Health Survey. J
Heal Med Informatics. 2014;5. doi:10.4172/2157-7420.1000152

6. Risk NCD, Collaboration F, WHO, Whelton H, Harrington JM, Crowley E, et al.
Boychart042. Lancet. 2016. doi:10.1159/000362780.Interpretation

7.	Islam MR, Rahman MS, Rahman MM, Nomura S, De Silva A, Lanerolle P, et al.
Reducing childhood malnutrition in Bangladesh: The importance of addressing socio-

economic inequalities. Public Health Nutr. 2020;23: 72–82.
doi:10.1017/S136898001900140X

8. Khan S, Zaheer S, Safdar NF. Determinants of stunting, underweight and wasting
among children < 5 years of age: Evidence from 2012-2013 Pakistan demographic and

health survey. BMC Public Health. 2019;19. doi:10.1186/s12889-019-6688-2

9.	International Institute for population Studies. National Family Health Survey - 4:

2015-16, India Fact sheet. National Family Health Survey - 4: 2015-16. 2016. pp. 1–6.
Available: <http://rchiips.org/NFHS/pdf/NFHS4/India.pdf>

10. Anon. Improving Child Nutrition - The achievable imperative for global progress.
United Nations Children’s fund. Division of Communication, UNICEF. 2013. doi:978-

92-806-4686-3

11. World Health Organization (WHO). Stunting | UNICEF. 2013 [cited 9 Nov 2017] p.

39. Available: <http://unicef.in/Whatwedo/10/Stunting>

12. WHO Multicentre Growth Reference Study Group. WHO Child Growth Standards.

Rev Chil pediatría. 2009. doi:10.4067/s0370-41062009000400012

13. Shiratori, Sakiko. Determinants of Child Malnutrition in Tanzania: a Quantile

Regression Approach. 2014 Annu Meet July 27-29, 2014, Minneapolis, Minnesota.
2014.

14. Oruamabo RS. Child malnutrition and the Millennium Development Goals: Much
haste but less speed? Arch Dis Child. 2015;100: S19–S22. doi:10.1136/archdischild-

2013-305384

15. Qu P, Mi B, Wang D, Zhang R, Yang J, Liu D, et al. Association between the Infant

and child feeding Index (ICFI) and nutritional status of 6- to 35-month-old children in
rural western China. PLoS One. 2017;12: e0171984.
doi:10.1371/journal.pone.0171984

16. Aber JL, Bennett NG, Conley DC, Li J. The Effects of Poverty on Child Health and
Development. Annu Rev Public Health. 1997;18: 463–483.
doi:10.1146/annurev.publhealth.18.1.463

17. PEDIATRICS COC. Poverty and child health in the United States. Pediatrics.
2016;137. doi:10.1542/peds.2016-0339

18. Gupta N, Zurn P, Diallo K, Dal Poz MR. Uses of population census data for
monitoring geographical imbalance in the health workforce: Snapshots from three

developing countries. Int J Equity Health. 2003;2: 1–12. doi:10.1186/1475-9276-2-1
19. Jukes M. The long-term impact of preschool health and nutrition on education. Food

and Nutrition Bulletin. Oxford University Press; 2005. pp. 23–37.
doi:10.1177/15648265050262S210

20. Bassole L, Bassole L, Bond S, Fapchamps M, Leibbrandt M, Nssah BE, et al.
“Opportunities and Challenges of Development for Africa in the Global Arena” A

Quantile Regression Analysis public infrastructure really matter? a Quantile
Regression Analysis. [cited 19 Mar 2018]. Available:
<http://repository.uneca.org/bitstream/handle/10855/15005/bib-58080.pdf?sequence=1>

21. Kumar A, Kumari D, Singh A. Increasing socioeconomic inequality in childhood
undernutrition in urban India: Trends between 1992-93, 1998-99 and 2005-06. Health

Policy Plan. 2015;30: 1003–1016. doi:10.1093/heapol/czu104

22. Corsi DJ, Mejía-Guevara I, Subramanian S V. Risk factors for chronic undernutrition

among children in India: Estimating relative importance, population attributable risk
and fractions. Soc Sci Med. 2016;157: 165–185. doi:10.1016/j.socscimed.2015.11.014

23. Mishra S, Pandey CM, Chaubey YP, Singh U. Determinants of Child Malnutrition in
Empowered Action Group (EAG) States of India. Stat Appl. 2015;13: 2454–7395.

24. Mekonnen T, Dune T, Perz J, Ogbo FA. Trends and determinants of antenatal care

service use in ethiopia between 2000 and 2016. Int J Environ Res Public Health.
2019;16. doi:10.3390/ijerph16050748

25. Messelu Y, Trueha K. Determining Risk Factors of Malnutrition among under-Five
Children in Sheka Zone, South West Ethiopia Using Ordinal Logistic Regression
Analysis. Public Heal Res. 2016;6: 161–167. doi:10.5923/j.phr.20160606.03

26. Li W, Liu E, BeLue R. Household water treatment and the nutritional status of
primary-aged children in India: Findings from the India human development survey.

Global Health. 2018;14. doi:10.1186/s12992-018-0356-7

27. Das S, Rahman RM. Application of ordinal logistic regression analysis in determining

risk factors of child malnutrition in Bangladesh. Nutr J. 2011;10: 124.
doi:10.1186/1475-2891-10-12422082256

28. Talukder A. Factors Associated with Malnutrition among Under-Five Children:
Illustration using Bangladesh Demographic and Health Survey, 2014 Data. Children.

2017;4: 88. doi:10.3390/children4100088

29. Srinivasan CS, Zanello G, Shankar B. Rural-urban disparities in child nutrition in

Bangladesh and Nepal. BMC Public Health. 2013;13. doi:10.1186/1471-2458-13-581 30. IIPS. National Family Health Survey (NFHS-3), 2005-06: India. Int Inst Popul Sci

Macro Int. 2007.

31. Singh S, Srivastava S, Upadhyay AK. Socio-economic inequality in malnutrition

among children in India: An analysis of 640 districts from National Family Health
Survey (2015-16). Int J Equity Health. 2019;18. doi:10.1186/s12939-019-1093-0

32. De Onis M. WHO Child Growth Standards based on length/height, weight and age.
Acta Paediatr Int J Paediatr. 2006;95: 76–85. doi:10.1080/08035320500495548

33. Bassolé L. Child Malnutrition in Senegal : Does Access to Public Infrastructure Really
Matter? A Quantile Regression Analysis. United Nations Economic Commission for
Africa; African Development Bank (2007-11). 2007. pp. 1–27.

34. Koenker R, Bassett G. Regression Quantiles. Econometrica. 1978;46: 33.
doi:10.2307/1913643

35. Koenker R, Hallock KF. Quantile regression. J Econ Perspect. 2001;15: 143–156.
doi:10.1257/jep.15.4.143

36. Cole TJ, Green PJ. Smoothing reference centile curves: The lms method and penalized
likelihood. Stat Med. 1992;11: 1305–1319. doi:10.1002/sim.4780111005

37. Yee TW. An Implementation for Regression Quantile Estimation. Compstat. Physica-
Verlag HD; 2002. pp. 3–14. doi:10.1007/978-3-642-57489-4_1

38. Yee TW. Quantile regression via vector generalized additive models. Stat Med.
2004;23: 2295–2315. doi:10.1002/sim.1822

39. Chen C. Growth charts of body mass index (BMI) with quantile regression.
Proceedings of the 2005 International Conference on Algorithmic Mathematics and

Computer Science, AMCS’05. 2005. pp. 114–120. Available:
<http://www.sas.com/statistics>.

40. Li Y, Graubard BI, Korn EL. Application of nonparametric quantile regression to body
mass index percentile curves from survey data. Stat Med. 2010;29: 558–572.
doi:10.1002/sim.3810

41. Ouyang Y, Wang H, Su C, Wang Z, Song Y, Xiao Y, et al. Use of quantile regression
to investigate changes in the body mass index distribution of Chinese adults aged 18-
60 years: A longitudinal study Biostatistics and methods. BMC Public Health.
2015;15: 278. doi:10.1186/s12889-015-1606-8

42. Sharaf MF, Mansour EI, Rashad AS. CHILD NUTRITIONAL STATUS in EGYPT: A
COMPREHENSIVE ANALYSIS of SOCIOECONOMIC DETERMINANTS USING
A QUANTILE REGRESSION APPROACH. Journal of Biosocial Science. 2019. pp.
1–17. doi:10.1017/S0021932017000633

43. Mani K. Determinants of Under-Five Mortality in Rural Empowered Action Group
States in India: An Application of Cox Frailty Model. Int J MCH AIDS. 2012;1: 60–

72. doi:10.21106/ijma.9

44. Vir SC. Chronic Child Undernutrition (Stunting) in India: An Overview of the

Problem, Determinants and Ongoing Efforts for Improving the Situation. Stat Appl.
2019;17: 221–234.

45. Khan REA, Raza MA. Determinants of malnutrition in Indian children: new evidence
from IDHS through CIAF. Qual Quant. 2016;50: 299–316. doi:10.1007/s11135-014-
0149-x

46. Mazumdar S. Determinants of inequality in child malnutrition in India: The poverty-
undernutrition linkage. Asian Popul Stud. 2010;6: 307–333.

doi:10.1080/17441730.2010.512763

47. Kandala NB, Madungu TP, Emina JBO, Nzita KPD, Cappuccio FP. Malnutrition

among children under the age of five in the Democratic Republic of Congo (DRC):
Does geographic location matter? BMC Public Health. 2011;11: 261.
doi:10.1186/1471-2458-11-261

48. Rashad AS, Sharaf MF. Economic Growth and Child Malnutrition in Egypt: New

Evidence from National Demographic and Health Survey. Soc Indic Res. 2018;135: 769–795. doi:10.1007/s11205-016-1515-y
