# Supplementary material for: Application of quantile regression to examine changes in the distribution of Height for Age (HAZ) of Indian children aged 0–36 months using four rounds of NFHS data
Source: PLoS One. 2022 May 27;17(5):e0265877. doi: 10.1371/journal.pone.0265877 (PMC9140296; doi:10.1371/journal.pone.0265877)
Supplement: Attachment — Submitted filename: Response to Reviewers.docx [file pone.0265877.s002.docx]

| **Journal Requirements** | **Author Response** |
| --- | --- |
| 1. Please ensure that your manuscript meets PLOS ONE's style requirements, including those for file naming. The PLOS ONE style templates can be found at  https://journals.plos.org/plosone/s/file?id=wjVg/PLOSOne_formatting_sample_main_body.pdf and  https://journals.plos.org/plosone/s/file?id=ba62/PLOSOne_formatting_sample_title_authors_affiliations.pdf | Thank you for highlighting the inconsistencies in the style requirements. We have now amended the manuscript to align with these guidelines. |
| 2. In your Data Availability statement, you have not specified where the minimal data set underlying the results described in your manuscript can be found. PLOS defines a study's minimal data set as the underlying data used to reach the conclusions drawn in the manuscript and any additional data required to replicate the reported study findings in their entirety. All PLOS journals require that the minimal data set be made fully available. For more information about our data policy, please see http://journals.plos.org/plosone/s/data-availability.  Upon re-submitting your revised manuscript, please upload your study’s minimal underlying data set as either Supporting Information files or to a stable, public repository and include the relevant URLs, DOIs, or accession numbers within your revised cover letter. For a list of acceptable repositories, please see http://journals.plos.org/plosone/s/data-availability#loc-recommended-repositories. Any potentially identifying patient information must be fully anonymized.    Important: If there are ethical or legal restrictions to sharing your data publicly, please explain these restrictions in detail. Please see our guidelines for more information on what we consider unacceptable restrictions to publicly sharing data: http://journals.plos.org/plosone/s/data-availability#loc-unacceptable-data-access-restrictions. Note that it is not acceptable for the authors to be the sole named individuals responsible for ensuring data access.  We will update your Data Availability statement to reflect the information you provide in your cover letter. | All the supporting data are available in the manuscript. We used secondary data “NFHS-1,2,3 and 4” for this study, which is publicly accessible at  <https://dhsprogram.com/what-we-do/survey/survey-display-355.cfm>. |
| 3. Please amend the manuscript submission data (via Edit Submission) to include author Thirupathi Reddy Mokalla. | Thank you. We have done the same |
| 4. Please amend either the abstract on the online submission form (via Edit Submission) or the abstract in the manuscript so that they are identical. | Thank you for highlighting the inconsistencies. We have now modified the abstract and it is identical at both the places |
| 5. Your ethics statement should only appear in the Methods section of your manuscript. If your ethics statement is written in any section besides the Methods, please move it to the Methods section and delete it from any other section. Please ensure that your ethics statement is included in your manuscript, as the ethics statement entered into the online submission form will not be published alongside your manuscript. | The study employed data from four rounds of publicly accessible National Family Health Survey (NFHS) data. NFHS has obtained ethical clearance before administering the surveys. Ethical approval is not needed for this study. The same is mentioned in page - **17.** |
| **Reviewer 1 Comments** | |
| **Reviewer Comments** | **Author Response** |
| Reviewer #1: The authors of "Application of Quantile Regression to examine changes in the distribution of Height for Age (HAZ) of Indian children aged 0-36 months using four rounds of NFHS data" submit an analysis that aimed to provide evidence of their objective, viz. "The objective of the study was to examine how the different determinants were heterogeneous in various percentiles of height for age (HAZ) distribution." They aptly lay out the background, methods, results and conclusions.  There are a number items that need to be addressed for which a de-identified file is attached that provides more comprehensive comments for the authors to improve the manuscript. Overarching in all of those is the following:  As the data are from a complex government survey, there are typically survey weights (aka design weights and post-stratification weights) applied to the data to account for survey design (design weights) or to adjust estimates to be generalizable to the population (post-stratification weights which will also contain the design weights). I do not see a discussion of these in the manuscript. If the authors are generalizing to the population, then post-stratification weights should be applied, otherwise, two things may happen:  1a. If post-stratification weights are not applied, results are not generalizable to the population;  1b. The design weights are meant to correct for the fact that complex surveys of this type are not a simple random sample. so that the observations are not independent and identically distributed (IID) so that statistical procedures that rely on this fact (viz. normal distributions) will not give correct estimates.  The saving grace here is that the beauty of quantile regression does not require IID and so this is a good reason to use quantile regression in this case [quantile regression has its origins to model data where unequal variance exists (Koenker, 2005)]. | **1a.** Stratification weights are not used in computing the quantile regression estimates.  Belasco, E.J., B. Chidmi, mentioned study results indicated that the two are not statistically different when using quantile regression, meaning both are consistent and implying the unweighted quantile regression is preferred because it is more efficient.  <https://scholarworks.montana.edu/xmlui/handle/1/8976>  **1b.** We thank the reviewer for the comment and we are completely in alignment with the reviewer’s view on quantile regression analysis which does not need the design weights because it does not follow the Gaussian/ Normal Distribution.  <http://www.econ.uiuc.edu/~roger/research/intro/rq.pdf> |
|  | |
| **Reviewer 2 Comments** | |
| **Reviewer Comments** | **Author Response** |
| 1. All background covers the stunting among under-five children, however under-three data was used in the analysis. It should be revised and prevalence and determinants for under-three data will be presented. | According to the National Family Health Survey (NFHS-1,2,3 and 4), the prevalence of stunted among children 0-36 months was 44.6 %, 45.5 %, 41.4%, and 36.6 %, respectively and the same were included in the page number **15.**  Introduction and background were changed accordingly |
| 2. Outcome is a composite one and age was included in HAZ definition. How was it also included in the model as an independent variable? | The age of the child has a strong non-linear impact on his/her nutritional status. To capture this effect, we included dummy variables that reflect different age categories.    <https://pubmed.ncbi.nlm.nih.gov/16087414/> |
| 3. How “size at the time of birth (small, average and large)” was defined in this study? | Since birth weight may not be known for  many babies in the field condition, the mother’s perception of the  baby’s size at birth was obtained for all births and classified size of the child at birth by NFHS questionnaire as follows: Very Large, Larger than Average,  Average, Smaller than Average and Very Small.  However, we have collapsed it into three categories which are  **Large** (containing Very large and Larger than Average),  **Average, and Small** (containing Smaller than Average and Very Small). |
| 4. Please mention the version of SAS software in the method section. | This analysis was performed using the SAS University Edition software and SAS® OnDemand for Academics. Same thing was included in the method section page number **19.**  <https://www.sas.com/en_us/software/university-edition.html> |
| 5. Please mention the level of statistical significance in the method section. | Statistically significant values p< 0.01, p<0.05, and p< 0.1 were include in the revised manuscript (page number **18**). |
| 6. Please add explanation about the approach for variable selection in the modelling process. | Thank you for this suggestion. This explanation is now added in the manuscript. please see page number **18**. |
| 7. Based on the comment 2, please clarify this interpretation “If the child's age increases, the coefficient values increase and these values are low in the lower quantiles compared to the upper quantiles) | Thanks for highlighting this we have corrected the typo graphical error in the page number **20** |
| 8. Confidence intervals for the effects should be presented. | we have added the confidence intervals to the estimates in the tables, changes can be found from table 2 to 6 |
| 9. Are presented effects in tables univariate or multivariable? | We have used the Multivariate quantile regression to estimate the coefficients on each percentile of the dependent variable (HAZ) |
| 10. Please mention how many babies were included in each percentile of response. | We have included total babies for each table (table 2 to 6).  We had mentioned the total no of children included for each table with gender separated values. because the quantile regression uses the full distribution for every quantile coefficient estimation.  **References**   1. <http://www.econ.uiuc.edu/~roger/research/rq/rq.pdf> 2. <https://link.springer.com/article/10.1186%2Fs40536-017-0048-4> 3. <https://journals.sagepub.com/doi/10.1177/0013164419837321> |
| 11. The manuscript requires grammatical and linguistic editing. | Thank you, we have edited and fixed the linguistic issues to the best of our ability. |
