# Supplementary material for: Application of quantile regression to examine changes in the distribution of Height for Age (HAZ) of Indian children aged 0–36 months using four rounds of NFHS data
Source: PLoS One. 2022 May 27;17(5):e0265877. doi: 10.1371/journal.pone.0265877 (PMC9140296; doi:10.1371/journal.pone.0265877)
Supplement: Attachment — Submitted filename: Responses to editor comments-06-03-2022..docx [file pone.0265877.s004.docx]

06^th^ March,2022

Dear Dr. Emanuele Giorgi,

Academic Editor.

PLOS ONE

Dear Sir,

We were pleased to have an opportunity to revise our manuscript entitled " **Application of Quantile Regression to examine changes in the distribution of Height for Age (HAZ) of Indian children aged 0-36 months using four rounds of NFHS data. (PONE-D-21-11573)**" to **PLOSONE**. We appreciate the time and effort that you and the reviewers and editor have dedicated to providing your valuable feedback on our manuscript. We are grateful to the editor and reviewers for their insightful comments on our paper. In the revised manuscript, we have carefully considered editors comments and suggestions. As instructed, we have attempted to briefly explain changes made in reaction to all comments. We reply to each comment in point-by-point fashion. We have the color-coded revised manuscript as text.

The responses to the concerns raised by editors/reviewers are below

The editors' comments were very helpful overall, and we are appreciative of such constructive feedback on our original submission. After addressing the issues raised, we feel the quality of the paper is much improved.

**Funding:** No specific funding has been received for this study.

Thanking You

Sincerely,

Dr. M. Vishnu Vardhana Rao

DIRECTOR, ICMR-NIMS

Min. of Health and Family Welfare

Govt. of India, New Delhi

**Responses to editor comments**

1) **Since I think the latter is more likely, then the paper has not illustrated and showed convincingly 1) what is the link between the research question and quantile regression and**

**Response:** The research question of the current study is to examine how the different determinants were heterogeneous in various percentiles of height for age (HAZ) distribution. In this study we explore the risk factors for childhood malnutrition in India using the quantile regression.

Traditional regression modelling approaches (linear/logistic regression) used to estimate the risk factors of childhood stunting often tend to oversimplify the complex interplay of a battery of risk factors through emphasizing a solitary risk and intervention. Recent studies in public health and epidemiological research called upon the application of a systems approach that focuses on examining the determinants of stunting in its entirety and seek to avoid the possibility of incorrect estimation of risk factors and programmatic interventions owing to any oversimplification. In addition, recent studies have also noted that the impact of risk factors at the lower tail of the distribution could be considerably different contrary to the population mean or at the higher end of the distribution. Therefore, in any population with a considerable degree of nutritional transition and disproportionate burden of anthropometric failure, it is critical to recognize and address the risk factors of stunting at the different ends of the spectrum and meticulously identify the actual effect of the risk factors at a different part of the distribution (i.e. lower tail, mean and upper bound of the distribution of z-score) to design and implement appropriate interventions. Quantile regression model quantiles of the outcome as a function of covariates and provides an opportunity to examine whether covariates have differential effects across the z-score distribution, particularly towards the lower tail. On the other hand, previous studies have largely examined the risk factors of stunting by applying logistic regression models for dichotomized versions of the Z-score (e.g. stunted vs. not stunted) or linear regression models for the continuous Z-score.

**2) why the research question cannot be answered using standard regression. I advise the authors to revise their manuscript substantially to expand more on the research question and the interpretational advantages of quantile regression.**

**For example, why should risk factors have different effects on the quantiles of the HAZ distribution?**

**Response:** We wish to study the different independent variables effect on the HAZ (dependent variable). In the linear regression we wish to study the mean affect on the dependent variable with the independent variables. Various studies have been identified in the prior literature, conducted in a wide range of developing nations and India, to explore the risk factors for child malnutrition by using a variety of methodologies and regression models. The linear regression model, multilevel or ordinal logistic regression models, and multilevel or ordinal logistic regression models were all used to analyse the nutritional status of children in the majority of the articles.

here we used the quantile regression over the standard regression (logistic regression and other methods), using the standard regression/ other regression we get one coefficient value and with their independent values only we study. The majority of the prior studies have been conducted with the HAZ score being dichotomized or categorise using logistic regression or linear regression, respectively. One significant drawback is that the dependent variable must be categorised according to certain criteria, which may reduce the statistical power of the odds ratio by a significant margin. When applied to a continuous response variable and a set of predictors, the linear regression (LR) model determines the association between them by using a conditional mean function. The LR technique, on the other hand, cannot be extended to non-central (other than mean) locations, and as a result, the complete conditional distribution of the outcome variable cannot be investigated. In practise, the model assumptions might be broken, and as a consequence, the estimated model may not accurately reflect the fluctuation of the response distribution in the real world.

But with the quantile regression we wish to study the HAZ effect on the different quaintiles (like 5,10, 25,70,75,90 and 95), with this we get the various coefficient value with respect to the quaintile , by using these values we can study the effect of the each independent variable on the different quiantile with independent variables.

Quantile regression model quantiles of the outcome as a function of covariates and provides an opportunity to examine whether covariates have differential effects across the z-score distribution, particularly towards the lower tail. On the other hand, previous studies have largely examined the risk factors of stunting by applying logistic regression models for dichotomized versions of the Z-score (e.g. stunted vs. not stunted) or linear regression models for the continuous Z-score.

3) **The paper also does not include references to statistical models that have been used to analyze HAZ data and does not provide any explanation as to why those methods cannot be used for the present study.**

**The revised version of the manuscript should therefore provide a more thorough review of previews statistical work in this field and comparisons should be carried out with the chosen method.**

Response: In manuscript we mentioned at page number 4 and reference numbers 26-32.

And also, below references were included in the manuscript

Reference:

1. Fenske, N., Kneib, T., Hothorn, T. (2011) Identifying Risk Factors for Severe Childhood Malnutrition by Boosting Additive Quantile Regression, Journal of the American Statistical Association, 106:494, 494-510, DOI: 10.1198/jasa.2011.ap09272
2. Fenske, N., Burns, J., Hothorn, T., Rehfuess, E.A. (2013) Understanding Child Stunting in India: A Comprehensive Analysis of Socio-Economic, Nutritional and Environmental Determinants Using Additive Quantile Regression. PLoS ONE 8(11): e78692. <https://doi.org/10.1371/journal.pone.0078692>
3. Banerjee K, Dwivedi LK (2020) Disparity in childhood stunting in India: Relative importance of community-level nutrition and sanitary practices. PLoS ONE 15(9): e0238364. <https://doi.org/10.1371/journal.pone.0238364>
